# Common Medical and Dental Problems of Older Adults: A Narrative Review

**DOI:** 10.3390/geriatrics6030076

**Published:** 2021-08-06

**Authors:** Alice Kit Ying Chan, Manisha Tamrakar, Chloe Meng Jiang, Edward Chin Man Lo, Katherine Chiu Man Leung, Chun-Hung Chu

**Affiliations:** Faculty of Dentistry, The University of Hong Kong, Hong Kong, China; dralice@hku.hk (A.K.Y.C.); manee626@hku.hk (M.T.); cmjiang@hku.hk (C.M.J.); hrdplcm@hku.hk (E.C.M.L.); kcmleung@hku.hk (K.C.M.L.)

**Keywords:** older adults, elderly, oral health, prevention, silver diamine fluoride, caries

## Abstract

The advancement of medicine has reduced the rate of mortality and older adult population is increasing. Among the 7,700,000,000 world population in 2019, 1 in 11 people were at the age of 65 or more. The population is expected to increase to 1 in 6 people by 2050. Older adults have degenerative changes that become more severe with age. This study used the World Health Organization’s websites and PubMed and Google Scholar databases to review current global oral and systemic health issues. Studies generally reported that many older adults have no regular dental checkup. Common oral diseases such as dental caries particularly root caries and periodontal disease are highly prevalent among them. These oral diseases are often interrelated with their systemic problems. A meta-analysis reported diabetes increases the incidence and progression of periodontitis by 86%. A decrease in salivary output is common among older adults having polypharmacy. A review reported the caries risk in older adults increases by 60% with low resting pH and low stimulated salivary flow rate. Many older adults suffer from dementia and depression which complicates the delivery of dental treatment. Proper oral hygiene practice and dental care at supine position are often difficult to be carried out if they have rheumatoid arthritis. With the increasing need of elderly dental care, dentists and other dental personnel should understand interlaced oral and general health in order to provide a successful dental care plan for older adults. The aim of this study is to give an overview of the common medical conditions and dental problems and their impacts on older adults.

## 1. Introduction

The world population was 7,700,000,000 in 2019 with 703,000,000 of older adults aged 65 or more. Driven by decreasing fertility rate and increasing life expectancy, the older adult population is expected to double to 1,500,000,000 in 2050. The proportion of older adult population will rise from 1 in 11 people to 1 in 6 people by 2050 [1]. Since older adults have degenerative changes which increase with age, the global disease burden will be increasing rapidly in the coming decades.

Chronic non-communicable diseases are becoming common because people have a longer life span. These chronic diseases together with the degenerative changes make older adults more vulnerable to oral diseases [2]. Systemic disease such as diabetes increases the incidence and progression of periodontitis by 86% [3] whereas other diseases such as rheumatoid arthritis make oral care practice difficult. Taking multiple medications for non-communicable diseases has adverse effects on oral health among which xerostomia is the most common problem. Xerostomia is further associated with other oral problems such as dental caries, periodontal disease and oral infections [4]. The increased risk of caries and periodontal disease may eventually lead to tooth loss. On the other hand, some oral diseases are interrelated with systemic diseases [5]. Diabetes and periodontal disease are known to have a bi-directional relationship [6] whereas studies implied the same relationship between rheumatoid arthritis and periodontal diseases [7,8]. Moreover, oral diseases and many chronic systemic diseases share the same risk factors such as unhealthy diet, tobacco use and alcohol consumption [9]. Use of tobacco is a risk factor for periodontitis, tooth loss and lung cancer while unhealthy eating habits can lead to dental caries, type 2 diabetes, coronal heart disease and stroke [10]. Oral diseases in older adults can adversely affect their general well-being and quality of life [11].

Since the chronic systemic diseases and their treatment can increase the risk of oral problems and interrelationship has been found among some systemic and oral problems, dental professionals should update with the current knowledge about common medical conditions of older adults and their impacts on the oral cavity. They should understand the relationship between oral and systemic diseases and the intricacies of changes related to aging to provide safe and effective dental care for older adults [12]. Dentists should also collaborate with other health professionals to modify dental treatment according to the elders’ needs and health conditions [13]. The aim of this review is to give an overview of the common medical conditions and dental problems and their impacts on older adults.

## 2. Impact of Common Systemic Diseases on Oral Health

The older population is increasing worldwide and they have degenerative changes that become more severe with age. Systemic diseases and the related medications make older adults more vulnerable to oral diseases [2]. In this article, the authors used information from the World Health Organization’s websites on current global oral and systemic health issues. They also used PubMed and Google Scholar databases to identify literatures in English including systematic reviews, meta-analysis, reviews and clinical studies on common medical and dental problems of older adults with no time restriction. Even though this study used the information provided by the World Health Organization and two of the most common databases to develop an updated global oral and systemic health issues, this study is a narrative review and the literatures included may not be comprehensive. However, this study identified six common systemic diseases which become more prevalent with age and they are common causes for morbidity and mortality.

### 2.1. Diabetes

Diabetes is the most common endocrine disorder in the older adult population. It is the ninth most common leading cause of death in 2019 which caused nearly 2,000,000 of death globally [14]. Its morbidity is mainly due to complications which include retinopathy, nephropathy, neuropathic foot ulceration and various cardiovascular diseases [15]. All these diabetic complications can be worsened if patients suffer from periodontal disease [16]. Diabetes is associated with multiple oral conditions such as periodontal disease, delayed wound healing, taste alteration and oral infections [6,17]. Salivary gland function is also impaired by increased glycosylated hemoglobin, neuropathy and microvascular abnormality of the microcirculation [17,18]. Hyperglycemia causes exaggerated inflammatory response which intensifies periodontitis [19,20]. A recent systematic review and meta-analysis found that diabetes increased the incidence and progression of periodontitis by 86% [3]. There is a bidirectional relationship between periodontal disease and diabetes [6]. The risk of periodontitis increases with uncontrolled diabetes whereas severe periodontitis adversely affects the glycemic control [6].

Studies showed that periodontal therapy together with good home oral health care was effective in improving the glycemic control for type 2 diabetic patients in 3 to 4 months [16]. It indicates the importance of good oral hygiene measures for this group of patients. All these studies indicate that periodontitis and diabetes have a bidirectional relationship and their respective control affects each other. Oral hygiene care is important for them not only to prevent periodontal disease but also for better glycemic control.

Diabetes also complicates tooth replacement. Diabetic patients are more susceptible to Candida infection [21]. Candida species from the oral cavity may migrate to upper gastrointestinal tract and lead to sepsis which requires a prolonged hospital stay and systemic antifungal drug [22]. Candida-associated denture stomatitis was more commonly found in denture-wearers with diabetes [23]. Underlying systemic diseases such as diabetes may mask the clinical symptoms of concomitant fungal infection of the oral mucosa in removable prostheses wearers [24]. Mycological examination may be used if in doubt [25]. Diabetic patients showed similar osseointegration rate as non-diabetic patients with implant survival rate of 97% [26]. However, they have 50% higher risk of having peri-implantitis and this highlights the importance of implant maintenance for them [27].

### 2.2. Hypertension

Hypertension is one of the most common cardiovascular conditions, which can cause cardiac failure and stroke if left untreated [18]. Over a billion people have the condition worldwide [28]. Oral manifestations of hypertension are mainly due to the side effects of anti-hypertensive medications including diuretics, calcium channel blockers, beta-blockers, angiotensin-converting enzyme inhibitors, alpha 2 blockers and angiotensin II receptor blockers. The main side effects of these drugs on the oral cavity are dry mouth, gingival hyperplasia, lichenoid reactions and taste alteration [18]. These side effects can be treated primarily by addressing the symptoms, e.g., increasing the water intake for patients with dry mouth or having localized periodontal therapy for those with gingival hyperplasia. Patients taking nifedipine are more prone to develop gingival hyperplasia than those taking amlodipine, hence dentists should consult their physicians to consider a change of medication in severe cases [29]. A systematic review and meta-analysis showed that periodontal disease increased the risk of hypertension with an odds ratio (OR) of 1.5; whereas the risk of hypertension increases with the severity of periodontal diseases with an OR of 1.64 in people with severe periodontitis [30]. However, the underlying mechanism is unclear.

Some studies found that intervention of periodontitis could help in controlling hypertension [31]. Hence, good oral hygiene not only improves oral health but also controls hypertension and hence reduces the risk of stroke and other cardiovascular diseases. Dentists should provide good pain control and avoid causing anxiety when treating patients with hypertension to minimize the risk of myocardial infarction and stroke attack during dental treatment. In the new ACC/AHA guideline published in 2017 [32], high blood pressure is now defined as systolic pressure > 130 mmHg (previously > 140 mmHg) or diastolic pressure > 80 mmHg. Under this new guideline, more patients will be diagnosed as hypertensive visiting the dental clinics. Blood pressure should be under control and measured before any dental procedures. American Dental Association recommends elective dental treatment can be performed for patients with systolic pressure < 160 mmHg and/or diastolic pressure < 100 mmHg while only emergency dental treatment is allowed for patients with systolic pressure between 160–180 mmHg and/or diastolic pressure between 100–109 mmHg with blood pressure monitoring every 10 to 15 min during the procedure [33]. If in doubt, dentists should seek advice from patient’s physician for necessary special precautions.

### 2.3. Rheumatoid Arthritis

Rheumatoid arthritis is a chronic inflammatory auto-immune destructive disease affecting multiple joints [34]. The older adult population with rheumatoid arthritis can be further divided into elderly-onset type which manifests after the age of 60 and young-onset type which starts at 20–40 years old [35]. This is the common cause for disability due to joint pain, inflammation-induced joint swelling and joint deformity caused by bone and cartilage destruction. These result in chronic pain and limited dexterity which make patients difficult to perform proper oral hygiene practice [35]. Rheumatoid arthritis is the common autoimmune disorder of secondary Sjogren’s syndrome, a slowly progressing immune-mediated disorder of exocrine glands that damages salivary glands and causes xerostomia. It also associates with oral complications such as periodontitis, temporomandibular joint dysfunction and its treatment can lead to methotrexate-induced oral ulceration. Moreover, the immunosuppressive effect of medications such as methotrexate and tumor necrosis factor (TNF)-α antagonists can further worsen the oral conditions [34].

Rheumatoid arthritis and periodontitis show some similarities as both are chronic inflammatory diseases leading to connective tissue destruction and share similar risk factors such as smoking and socioeconomic status [34]. A study showed that periodontal disease increased the risk of having rheumatoid arthritis by 69% and it may be due to their shared inflammatory pathway [7]. Short term clinical trials found that non-surgical periodontal therapy can reduce disease activity of rheumatoid arthritis by 18% [36]. The prevalence of degenerative temporomandibular joint disease in people with rheumatoid arthritis ranged from 45% to 93% [37], which is presented as limited mouth opening, tenderness, muscle spasm and clicking sound/crepitus. This further complicates oral hygiene care [34]. Xerostomia is commonly found in people with rheumatoid arthritis. Secondary Sjogren’s syndrome, causing dry eye and mouth, may occur in 30–50% of people with rheumatoid arthritis. Reduced salivary flow and the changes in salivary composition increase the risk of dental caries and periodontal disease [34]. Dental treatment should be carried out in a comfortable position with the head and neck well supported [38]. As rheumatoid arthritis sufferers have increased risk of caries and periodontitis, preventive measures such as regular topical fluoride are extremely important for them [34].

### 2.4. Alzheimer’s Disease

Alzheimer’s disease is a progressive neurodegenerative disease and is the most common form of dementia in older adults [39]. Its main feature is deterioration of cognitive function, which is irreversible in nature, affecting memory, coordination, emotional control, behavioral function and motor skills [39,40]. Oral hygiene in Alzheimer’s patients is usually poor due to the impaired cognitive function, memory loss and decreased motor skills. This leads to oral problems such as caries, periodontal disease and denture-induced stomatitis. These risks are further aggravated by xerostomia induced by Alzheimer’s drugs [39]. A systematic review showed that people with periodontal disease had increased risk of having Alzheimer’s diseases with the OR of 1.69 [41] due to the systemic effect of the increased bacterial load and inflammation process [42]. However, further studies are needed to investigate this association and the underlying mechanism. 

Dental management depends on the disease stage and it should be prevention oriented, with long lasting outcome and easy maintenance. At the early stage of the disease, preventive measures are essential as patients are still competent to perform their own oral hygiene to prevent oral diseases and maintain self-care. Use of novelty devices such as an electric toothbrush and a water flosser as well as innovative strategies such as 5S methodology to develop oral hygiene habit can be considered in this group of patients [43,44]. Progression of the disease makes the patients more uncooperative and agitated and hence complicates the treatment in the advanced stage [40]. Therefore, initiating preventive measures and oral rehabilitation in patients with Alzheimer’s disease at the early stage of disease is important [39,40]. In the later stage, the family members or caregivers should be encouraged to learn and assist in the maintenance of their oral hygiene [40].

### 2.5. Parkinson’s Disease

Parkinson’s disease is a progressive neurodegenerative disorder characterized by motor and non-motor symptoms that affects the individual physically, emotionally and cognitively [45]. Motor symptoms include resting tremor, bradykinesia, postural instability and rigidity [45] while non-motor symptoms include apathy, cognitive disorder, sleep disturbance and orthostatic hypotension [45,46]. Depression and anxiety are seen in around 50% of the older adults with Parkinson’s disease [46]. Motor impairments due to tremors and rigidity of the orofacial musculature cause difficulty in tooth brushing which is the primary risk factor for oral diseases such as caries and periodontal disease [47]. The prevalence of periodontitis in Parkinson’s patients has been reported to be 75%, with most of them in the severe form [47]. Patients with periodontitis had an increased risk of developing Parkinson’s disease by 43% [48] but further studies are needed to understand their interrelationship. Involuntary jaw movement may cause temporomandibular joint discomfort, cracked teeth, tooth wear, orofacial pain and dysphagia [46]. Anti-Parkinson medications and anti-depressants can induce xerostomia [45] and hence increase the risk of dental caries and periodontal disease [4]. Parkinson’s disease is progressive in nature and therefore dental treatment should be considered at the early phase of the disease [46].

### 2.6. Depression

Depression is the most common mental illness that affects older adults and is the third most common cause of disability worldwide [49,50]. Depression in older adults can be caused by suffering of medical illness, physical disabilities, loneliness, financial problems and unpleasant life events such as the death of spouse, while sometimes without any obvious cause [50]. It adversely affects mood, concentration, way of thinking, sleep and appetite. It also causes patients a lack of motivation, loss of interest and having low self-esteem. In addition, it can exaggerate pain perception and lower the pain tolerance of the patients [50]. Lack of energy and motivation change their behavioral habits such as neglecting oral health care, developing smoking habit and shifting to a cariogenic diet [49]. Anti-depressants, especially tricyclic anti-depressants, cause xerostomia. These changes increase the risk for dental caries and periodontal disease [49]. A recent systematic review indicated that depression increased the risk of oral diseases including dental caries (OR 1.27), tooth loss (OR 1.31) and edentulism (OR 1.17). Conversely, edentulism (OR 1.28) and periodontal disease (OR 1.73) increased the risk of having depression [49]. This signifies the importance of preventive measures for patients with depression and also the psychological impact of poor oral health on them. Dentists should be supportive, patient and non-judgmental on managing older adults with mental illness [50]. Educating patients and their caregivers about proper oral hygiene practice together with preventive measures at the early stage of disease is essential [50]. 

## 3. Oral Health Conditions with Aging

Oral health is one of the essential components to “healthy aging” as it affects the individuals’ overall health and quality of life [51]. According to the declaration of the FDI (World Dental Federation) in 2016, oral health is important for speech, smile, taste, chewing and social communication and hence affects overall health, well-being and quality of life [11]. People are retaining more teeth for lifetime because of the advancement in the oral health services and emphasis on prevention. It results in increasing number of dentate older adults facing dental problems [51]. Periodontal disease, dental caries, tooth loss, xerostomia and oral precancerous and cancerous conditions are the common oral health problems in older adults causing major public health issue in our society [2,52].

### 3.1. Periodontal Disease

Periodontal disease is a chronic inflammatory disease cumulating throughout the life and becomes irreversible if it progresses to periodontitis. It affects tooth supporting structures causing gingival recession, alveolar bone resorption, tooth mobility and eventually tooth loss [53]. Gingival recession is a risk factor for root caries which is highly prevalent in the older adult population. Mobility of teeth and tooth loss diminish masticatory efficiency resulting in great psychological stress, difficulties in daily functioning and reduced quality of life [12].

Periodontal disease affects about 45–50% of adults in its mildest forms of periodontitis, rising to over 60% in older adults aged 65 or above [54]. A review showed that 11% of the world population suffered from severe periodontitis [55]. The prevalence of periodontitis for people in US at the age of 65 or more was 66%, and among them up to 23% had severe periodontitis [56]. Periodontal disease is not a mere consequence of aging but induced by dental biofilm and therefore proper oral health care can prevent its occurrence. The risk factors for periodontitis are similar for all age groups but are more aggravated in the older adults. This is mainly due to decreased immunity, systemic diseases such as diabetes, reduced manual dexterity and diminished visual acuity [52], and its systemic impact in the older adult population is more profound. There is increasing evidence indicating an association between periodontal disease and several comorbidities, for instance, diabetes, cardiovascular diseases, rheumatoid arthritis, Alzheimer’s disease and Parkinson’s disease due to their shared inflammatory pathway and improving periodontal condition could improve these medical conditions [57]. Hence, having good oral hygiene can improve oral health as well as systemic health in older adults.

### 3.2. Dental Caries

Dental caries remains the major public health issue globally and is the fourth most expensive chronic disease for treatment according to the World Health Organization [58]. It induces pain, infection and eventually tooth loss if left untreated and negatively impacts general well-being and quality of life [2]. In 2010, there were 2,400,000,000 adult people worldwide affected by untreated caries. There are three peaks during the lifetime when prevalence of caries is high, which are at ages 6, 25 and 70. This indicates that older adults are at risk of caries development [59]. A decrease in salivary output is common among older adults having polypharmacy. A review reported the caries risk in older adults increases by 60% with low resting pH and low stimulated salivary flow rate [60]. There is a vast difference in caries prevalence in older adults between developed and developing places. In developing countries such as India, majority of older adults had caries with a prevalence of 82% while in developed countries such as Germany, the prevalence of caries in older adults was almost 30% [56,61]. However, data for the prevalence of caries status in the older population are scarce and most were updated decades ago. There is an urgent need to investigate the current caries status in this population in order to design appropriate health policies. Root caries is more prevalent in the older adult population because of root exposure after gingival recession. The exposed rough and irregular cementum is more susceptible to plaque retention and demineralization. Other risk factors include old age, low socio-economic status, tobacco use and poor oral hygiene [62]. As older adults are retaining more teeth for longer, more root caries would be expected in our growing older adult population and hence the demand and burden for its treatment would be increasing [63]. Conventional dental care is often difficult for older adults due to constraints such as accessibility and affordability. Hence, it is important to have a non-invasive and cost-effective method for caries control and prevention for them.

Fluoride therapy is effective for caries prevention [64]. Frequent dental check-ups, professional tooth cleaning and patient education on oral hygiene instruction and dietary advice are also beneficial. High dose fluoride toothpaste is another fluoride agent that can be used to manage dental caries in the older adult population [64]. Silver diamine fluoride (SDF) has been recommended as a cost-effective and non-invasive caries arrest agent [65]. A systematic review supports using SDF on root caries prevention in the older adults [66]. In 2018, American Dental Association stated that SDF was a non-restorative treatment for caries arrest on permanent teeth and, in 2020, recommended its use for caries management [67]. More well-designed clinical trials should be conducted to provide more information on their clinical uses in older adults for caries management.

### 3.3. Edentulism

Tooth loss is the endpoint of severe dental caries and periodontitis which eventually leads to complete edentulism [52]. Tooth loss affects the general oral health [68]. After tooth extraction, the adjacent teeth may drift towards each other and the opposing tooth may over-erupt [69,70], loss of proximal contacts and increase in interproximal space may increase the chance of food trapping and hence caries and periodontal disease. Missing front teeth affects aesthetics and speech while multiple missing teeth affects chewing function [68]. Tooth loss resulted in edentulism or lack of functional dentition affects nutritional intake and poses an increased risk of malnutrition by 21% [71]. Tooth loss can provoke negative impacts on social status, self-esteem and oral health-related quality of life [72]. In addition, a review reported that patients with tooth loss had an increased risk of having depression by 28% [49]. Older adults with multiple missing teeth had a 55% higher risk of having dementia while those with more teeth would have 50% reduced risk of having dementia [73,74].

### 3.4. Xerostomia

Saliva plays an important role in maintaining oral health. It helps in chewing, lubrication of food, deglutition, taste sensation and speech. It also helps to prevent dental caries and other oral infections due to its antimicrobial, cleansing and buffering properties. Xerostomia is a common oral problem in older adult population with a prevalence of 33% [75]. Xerostomia is a subjective feeling of oral dryness while hyposalivation is an objective finding of decreased salivary production [76]. With aging, both major and minor salivary glands undergo degenerative changes with atrophy of the acinar tissues and hence the salivary output is decreased [77]. Systemic factors including diabetes, alcoholic cirrhosis, auto-immune diseases such as Sjogren’s syndrome and rheumatoid arthritis as well as anxiety and stress can affect the salivary gland function. Medication uses such as diuretics, sedatives, antihypertensives and antiparkinsonian drugs are associated with xerostomia and salivary gland hypofunction [4]. The effect is medication type-dependent with urological medication imposing the greatest risk (OR 5.91) and is increased with the number of medications taken [78]. Patients with xerostomia have increased risk of dental caries and periodontal disease, difficulty in speaking and swallowing, burning mouth syndrome, taste alteration and denture related problems such as soreness, ulcers and Candida infection [4]. Preventive measures such as fluoride application, oral hygiene instruction, dietary advice and use of saliva substitutes are useful for them [4].

### 3.5. Oral Precancerous and Cancerous Lesions

Oral mucosa is the lining of the oral cavity which protects against local irritants. However, oral epithelium becomes thinner and non-resilient with age [53]. The mucosa becomes permeable to toxic substances and less disease-resistant. This predisposes patients to cancerous and precancerous lesions which can be serious and life threatening [53]. Cancer of oral cavity, lips and pharynx is the eighth most common cancer globally. It mainly affects people aged 65 years or above and is more common in under-developed countries than developed countries [52]. The most common form of oral cancer in older adults is squamous cell carcinoma arising from the lining of the oral mucosa [53]. The treatment in general are surgical excision, radiotherapy and chemotherapy [52]. Radiotherapy often causes irreversible salivary gland damage with reduced saliva secretion when the salivary glands are included in the irradiated field. Preventive measures for the aftercare of these patients are crucial to reduce the risk of dental caries, periodontal disease and other oral infection.

### 3.6. Tooth Wear

In the course of lifetime, there are many anatomical and histological changes in tooth structure due to chemical attack and mechanical forces, hence tooth wear is commonly found in older adult population [79]. Prevalence of tooth wear increases with age from 3% at the age of 20 years to 17% at the age of 70 years [80]. Tooth wear can be caused by attrition, abrasion, erosion and any combination of these [81]. Attrition is a physiological process due to tooth-to-tooth contact. It is presented as flat and smooth occlusal and incisal surfaces. Abrasion is pathological wear due to extrinsic mechanical forces which results in a notch at the cervical area with rough and sharp edge of the enamel. Erosion appears as shiny and glossy surface with rounded edge due to the chemical dissolution. The loss of tooth structure is commonly seen on the palatal or labial surfaces depending on the source of erosion which can be either extrinsic or intrinsic [81,82]. Anterior teeth are more affected by tooth wear than posterior teeth [81]. Clinical presentations may include sharp tooth edges, dentine hypersensitivity, shortened clinical crown height and reduced lower face height. In severe conditions, it may expose the pulp and leads to pulpitis and pulpal necrosis [79,81]. Dietary modification and fluoride application are found to be effective in the control of tooth wear and alleviation of the symptoms [83].

## 4. Conclusions

In conclusion, oral and general health are interrelated and sometimes in a bidirectional relationship. Diabetes, hypertension, rheumatoid arthritis, Alzheimer’s disease, Parkinson’s disease and depression are common diseases that become more prevalent with age. These systemic diseases and their related medications make older adults more vulnerable to oral diseases such as periodontal disease, dental caries and even oral precancerous and cancerous lesions. Older adults have degenerative changes that become more severe with age. Many of them have substantial medical and dental problems. They may suffer from xerostomia and tooth wear. Their oral diseases and conditions are often interrelated with their systemic problems. Dental professionals should update with current knowledge and skills in geriatric dentistry to cope with the increasing need of elderly dental care.
